# Integrated analysis of metastasis-related circRNA-miRNA-mRNA ceRNA regulatory network involved in breast cancer

**DOI:** 10.1016/j.clinsp.2025.100826

**Published:** 2025-11-29

**Authors:** Hongming Song, Mingkai Gong, Wenfeng Li, Chen Liu, Haiyan Hu, Zhongliang Ma, Xiangping Liu, Yuanxiang Wang, Bing Du, Haibo Wang

**Affiliations:** a Breast Disease Center, The Affiliated Hospital of Qingdao University, Qingdao, Shandong, China; b Medical Research Center, The Affiliated Hospital of Qingdao University, Qingdao, Shandong, China; c Qingdao Medical College, Qingdao University, Qingdao, Shandong, China

**Keywords:** Circrna, Cerna, Network, Breast cancer, hsa_circ_0089131

## Abstract

**Study guidelines:**

This study followed the STROBE Statement.

**Background:**

Breast cancer persists as a major cause of cancer-related mortality among women.

**Methods:**

The expression data of circRNA (GSE101123, GSE111504, GSE182471, GSE165884), miRNA (GSE154255), and mRNA (GSE52194) in breast cancer were downloaded from the GEO databases. The sponge miRNA and mRNA corresponding to the DEcircRNA were predicted through CircInteractome, circBank databases, and StarBase. The enrichment and annotation of all the miRNAs were analyzed through the miEAA database. The circRNA-related ceRNA network of breast cancer was constructed by the STRING database and Cytoscape software. DAVID database was used to analyze the GO and KEGG enrichment of DEmRNAs. RT-qPCR, StarBase database, and GEPIA database were utilized to validate the expression of hsa_circ_0089131, miR-378a-3p, and TP53 in breast cancer.

**Results:**

The four circRNA-associated GEO databases overlapped two metastasis-related DEcircRNAs, including hsa_circ_0001936 and hsa_circ_0089131. After the enrichment and annotation of all the DEcircRNA-related miRNAs overlapped with GSE154255, a total of 60 breast cancer-associated miRNAs were obtained. Finally, the ceRNA network with 2 circRNAs, 44 miRNAs, and 122 mRNAs was constructed and visualized. The breast cancer-related mRNAs are enriched in various pathways, especially AKT/PI3K and MAPK signaling pathways. The expression of hsa_circ_0089131 (upregulated), miR-378a-3p (downregulated), and TP53 (upregulated) was validated in breast cancer tissues and showed a correlation.

**Conclusion:**

The ceRNA regulatory network related to the metastasis and prognosis of breast cancer was constructed, which included hsa_circ_0089131, miR-378a-3p, and TP53. This study provides a bioinformatics basis for elucidating the molecular mechanism of metastasis and the prognosis of breast cancer.

## Highlights

• Four GEO databases overlapped DEcircRNAs hsa_circ_0001936 and hsa_circ_0089131.
• Breast cancer-related network with 2 circRNAs, 44 miRNAs, 122 mRNAs was constructed.
• Circ_0089131-miR-378a-3p-TP53-PI3K-Akt/MAPK pathway ceRNA network was constructed.

## Introduction

According to the latest common cancer statistics in the US in 2025, breast cancer alone made up 32 % of all newly diagnosed cancer cases, which is the most prominent.^1^ Although China has a relatively low incidence of breast cancer in contrast to other Asian countries and the US, rapid urbanization and lifestyle changes may contribute to an increasing number of breast cancer cases in China.^2^ Despite early diagnosis, radical surgery for non-metastatic patients, and adjuvant therapy having substantially improved the survival and prognosis of breast cancer patients, the current treatment for patients with metastatic disease still has limitations, and breast cancer is still one of the leading causes of cancer-related death in women.^3^ With the rapid development of bioinformatics methods and high-throughput sequencing techniques, it is of great significance to continuously explore the bioinformatics basis of the occurrence and progression of breast cancer and then strive to maximize the early diagnosis and treatment effect.

Non-coding RNA (ncRNA), including circular RNA (circRNA), lncRNAs, and microRNAs (miRNAs), is a group of RNA molecules that lack the capacity to encode proteins but with regulatory functions in diseases.^4^^,^^5^ As an important class of ncRNA, circRNA plays a crucial regulatory role in the occurrence, metastasis, resistance to chemotherapy, and radiation in cancers, and may even play an indispensable role in the molecular typing of breast cancer.^6^^,^^7^ The biological functions of circRNAs in eukaryotes include serving as miRNA sponges, regulating gene transcription, and interacting with proteins.^8^ In addition, circRNAs can act as competing endogenous RNAs (ceRNA) to participate in the progression of breast cancer through competitively binding miRNA to eliminate the inhibitory effect of miRNA on downstream mRNA, and affect the regulation of tumor progression.^9^ For instance, increased circKIF4A in Triple-Negative Breast Cancer (TNBC) competed for binding to miR-637 to regulate STAT3 expression, then promoted brain metastasis in TNBC, thereby serving as a therapeutic target.^10^ The research on circRNA suggests that it is expected to be a predictor for breast cancer diagnosis, prognosis, and a therapeutic target, but the present research is far from enough, and it still needs to continue to be explored.

Currently, more than 1 million samples are available in the GEO database. In addition, GEO not only provides public data for researchers but also provides tools to help users identify, analyze, and visualize relevant genomic data of interest. This study aimed to identify the Differential Expressed (DE) circRNAs, miRNAs, and mRNAs in breast cancer patients by analyzing the microarray data from public databases and exploring their functions. Further, the ceRNA network was constructed to provide some basis for circRNA in breast cancer. Then, the expression circ_0089131-miR-378a-3p-TP53 axis was validated.

## Materials and methods

### Identification of metastasis breast cancer-related microarray profiles

To explore the breast cancer-associated circRNA profiles, the microarray profiling analysis datasets of circRNAs in breast cancer (GSE101123, GSE182471, GSE165884, GSE111504) were analyzed. The GSE101123 dataset (Arraystar Human CircRNA microarray V1) included 10 breast cancer tissues and 3 mammary gland tissues. GSE182471 datasets and GSE165884 (Arraystar Human CircRNA microarray V2) included 5 pairs and 4 pairs of breast cancer tissue and adjacent non-tumor tissue samples, respectively. GSE111504 dataset assessed the expression profiles of circRNAs in parental MDA-MB-23A (231-PAR), isogenic Brain Metastatic cells (231-BM6), Lung Metastatic cells (LM2), and bone metastatic cells (1833) by Arraystar Human CircRNA microarray V2.

In addition, differential molecular subtypes (luminal A, B, and HER2-positive and triple-negative breast cancer) of breast cancer-related DEmiRNAs profiles were analyzed using the GSE154255 dataset, in which 3 pairs of HER2 cancer tissues and adjacent non-tumor tissues, 2 pairs of luminal A cancer tissues and adjacent non-tumor tissues, 2 pairs of luminal B cancer tissues and normal tissues, and 3 pairs of TNBC cancer tissues and adjacent non-tumor tissues. The three breast cancer subtypes (TNBC, non-TNBC, and HER2-positive)-related DEmRNA was analyzed using the GSE52194 dataset. The information about these GEO datasets is listed in Table 1.

**Table 1:** The information of the GEO datasets used in this study.

Datasets	Profile	Samples	Platforms
GSE101123	circRNA	10 breast cancer tissues, 3 mammary gland tissues	GPL19978
GSE182471	circRNA	5 breast cancer tissues, 5 adjacent non-tumor tissues	GPL21825
GSE165884	circRNA	4 breast cancer tissues, 4 adjacent non-tumor tissues	GPL21825
GSE111504	circRNA	Parental 231-PAR, brain metastatic 231-BM6, lung metastatic LM2, bone metastatic cells 1833	GPL21825
GSE154255	miRNA	3 pairs HER2 cancer tissues and non-tumor tissues	GPL18402
		2 pairs luminal A cancer tissues and non-tumor tissues	
		2 pairs luminal B cancer tissues and normal tissues	
		3 pairs TNBC cancer tissues and non-tumor tissues	
GSE54194	mRNA	17 breast tumor samples (6 TNBC, 6 non-TNBC, and 5 HER2-positive)	GPL18187
		3 normal human breast organoids (epithelium) samples (NBS)	

The interactive web tool GEO2R, based on *R* packages was utilized to identify DEcircRNAs, DEmiRNAs, and DEmRNAs in breast cancer. The differentially expressed RNAs were obtained on the basis of the condition of p-value < 0.05 and |log FC| > 0.6.

### Downstream miRNAs of circRNAs prediction and functional enrichment analysis

The four circRNA-associated GEO databases overlapped two metastasis-related DEcircRNAs. Then, their downstream miRNAs were predicted using CircInteractome, Starbase, and circBank databases. All the obtained miRNAs were enrolled in the functional enrichment and annotation analysis using the miRNA Enrichment Analysis and Annotation Tool (miEAA; https://ccb-compute2.cs.uni-saarland.de/mieaa/) with miRNA enrichment analysis (GSEA) analysis type. Based on the enrichment and annotation results, breast cancer-related miRNAs overlapped with the DEmiRNAs from the GSE154255 dataset.

### ceRNA network construction

The target mRNAs were predicted using the StarBase database. Then the breast cancer-related miRNAs that are invalid were excluded. The target mRNAs with TDMD > 1 were enrolled in the following analysis. With the help of STRING (high confidence 0.700, hide disconnected nodes in the network) and Cytoscape software (top 15 nodes ranked by MCC), the top 15 hub genes of each miRNA were obtained, and all the target mRNAs overlapped with the DEmRNAs from GSE54194 (p < 0.05, |logFC| > 1) to obtain the downstream mRNAs. The Cytoscape software was utilized to visualize the ceRNA network.

### Protein-protein interaction network

Most proteins carry out their biological functions through interactions with themselves or other molecules. Protein-Protein Interaction (PPI) network represents the interaction between relevant proteins within cells and can gain biological insights into protein functions, disease prevalence, and the development of therapies.^11^ The PPI network was conducted utilizing the STRING database with a high confidence of 0.700, and hidden disconnected nodes in the network.

### GO and KEGG enrichment analysis

The Database for Annotation, Visualization, and Integrated Discovery (DAVID) offers a wide-ranging collection of functional annotation tools to help decipher the biological significance underlying extensive gene lists.^12^ The DAVID tool was used to analyze the GO and KEGG enrichment of these downstream targets. The bioinformatics online tool was utilized for visualization of GO and KEGG enrichment.

### Sample collection

Fifty pairs of breast cancer tissues and adjacent non-tumor tissues were gathered from breast cancer patients who received surgical operations in the Breast Disease Center of the Affiliated Hospital of Qingdao University (Qingdao, China) from July 2020 and July 2022. All tissue samples included in the study were diagnosed by two pathologists. The tissue samples were kept in liquid nitrogen until use. Every patient signed the written informed consent form. Moreover, this study received approval from the Ethics Committee of the Affiliated Hospital of Qingdao University (No. QYFY-WZLL-30695).

### Isolation of total RNA and RT-qPCR

Total RNA was extracted from tissues utilizing Trizol reagent (Invitrogen) and its concentration was determined by Nanodrop 2000 (Thermo Fisher Scientific). The cDNAs were reverse transcribed using the QuantiTect reverse transcription kit (Qiagen) for circRNA and mRNAs, and the miScript Reverse Transcription Kit (Qiagen) for miRNAs. The quantitative PCR was performed with the QuantiTect SYBR Green PCR Kit or miScript SYBR Green PCR Kit. Relative expressions were determined by applying the 2^-ΔΔCt^ method, where GAPDH or U6 was used as the control.

### Expression and prognostic value validation

The StarBase database was used to identify the expression of miR-378a-3p in breast cancer. The GEPIA2 database was used to verify the expression and prognostic value of TP53 in breast cancer.

### Statistical analysis

All data were obtained from at least three replicates and expressed as the mean ± SD. Student *t*-test was used to compare the differences between groups. Pearson correlation analysis was performed to analyze the correlation between hsa_circ_0089131, miR-378a-3p, and TP53. Statistical significance is represented by a p-value less than 0.05.

## Results

### DEcircRNAs in breast cancer

**Fig. 1:**
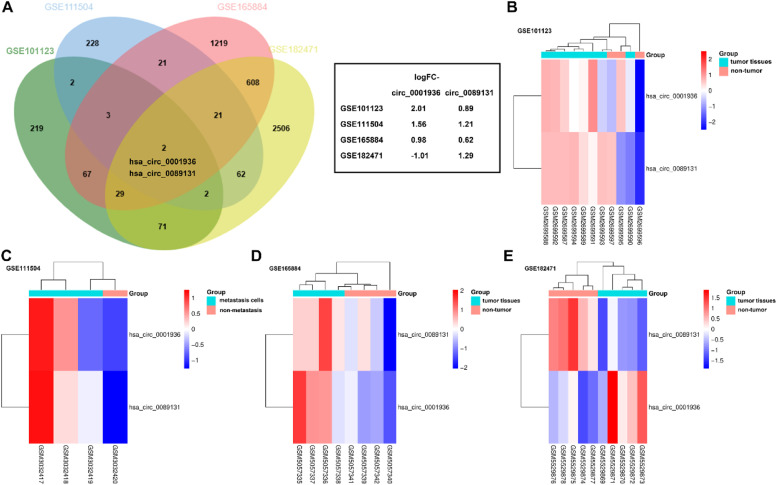
Differential expressed circRNAs (DEcircRNAs) in breast cancer. (A) DEcircRNAs in GSE101123, GSE165884, and GSE182471 overlapped with metastasis related DEcircRNAs in GSE111504 dataset. (B‒E) The heatmap of hsa_circ_0001936 and hsa_circ_0089131 expression in GSE101123 (B), GSE111504 (C), GSE165884 (D), and GSE182471 (E).

The DEcircRNAs (adjusted p < 0.05, |logFC| > 0.6) were identified from the datasets with accession numbers GSE101123, GSE182471, GSE165884, and GSE111504 that were analyzed by GEO2R. Then, the DEcircRNAs from GSE101123, GSE182471, and GSE165884 were overlapped with the metastasis-related DEcircRNAs from the GSE111504 datasets. Both hsa_circ_0001936 and hsa_circ_0089131 were obtained after preliminary screening and interaction by VENN (Fig. 1A). The expression of hsa_circ_0001936 and hsa_circ_0089131 was displayed in a heatmap among the four GEO datasets (Figs. 1B‒E). The hsa_circ_0089131 showed a consistent upregulated trend in breast cancer and associated with metastasis (Fig. 1).

### CircRNA-associated miRNAs function enrichment

**Fig. 2:**
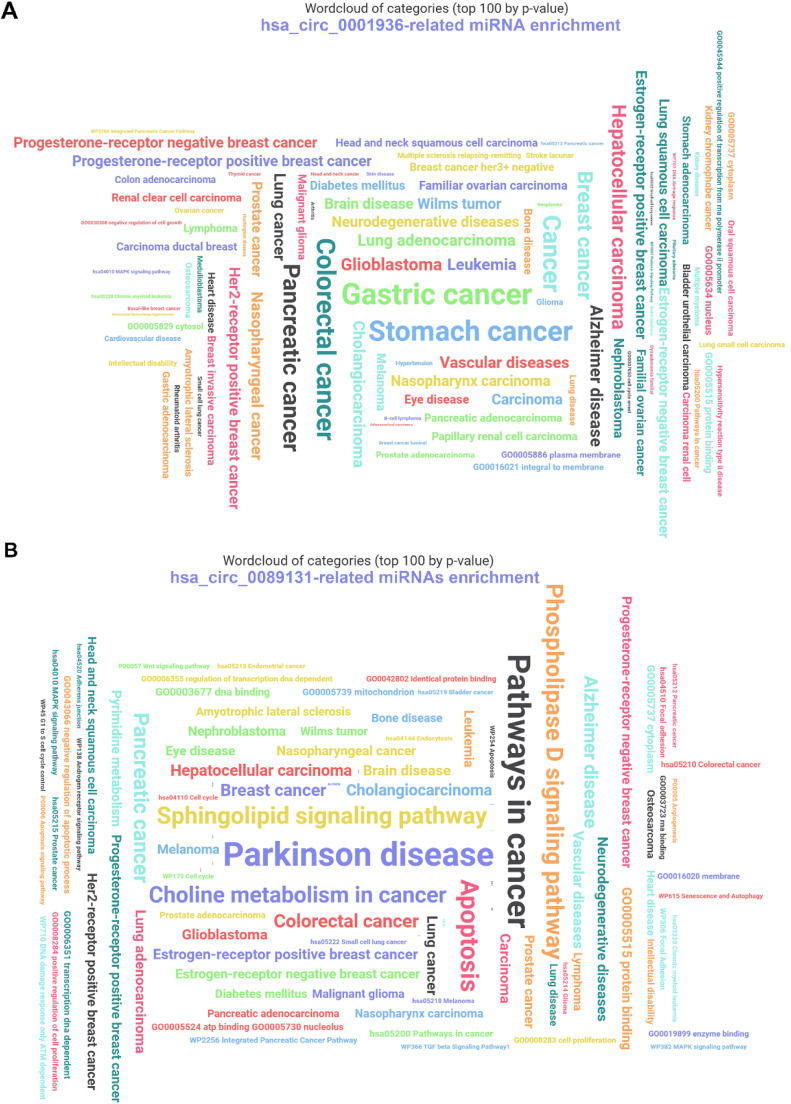
The functional enrichment and annotation analysis of downstream miRNAs of hsa_circ_0001936 and hsa_circ_0089131 were analyzed using the miEAA online database. (A) The function enrichment and annotation of hsa_circ_0001936-related miRNAs. (B) The annotation of hsa_circ_0089131-related miRNAs.

The downstream miRNAs of hsa_circ_0001936 and hsa_circ_0089131 were predicted using CircInteractome, Starbase, and circBank databases. The three databases obtained hsa_circ_0001936 downstream miRNAs (*n* = 184) and hsa_circ_0089131-related downstream miRNAs (*n* = 247). All the obtained miRNAs (invalid miRNAs were excluded) were enrolled in the functional enrichment and annotation analysis using the miEAA online database with miRNA enrichment analysis (GSEA) analysis type. The data show that hsa_circ_0001936 downstream miRNAs are enriched in various diseases, including differential molecular subtypes of breast cancers (Fig. 2A). hsa_circ_0089131-related downstream miRNAs have many functions, such as regulation of cell proliferation and apoptosis, DNA, RNA, and protein binding, as well as angiogenesis. In addition, these miRNAs are involved in various diseases, including differential molecular subtypes of breast cancer (Fig. 2B).

### CircRNA-miRNA-mRNA ceRNA network construction

**Fig. 3:**
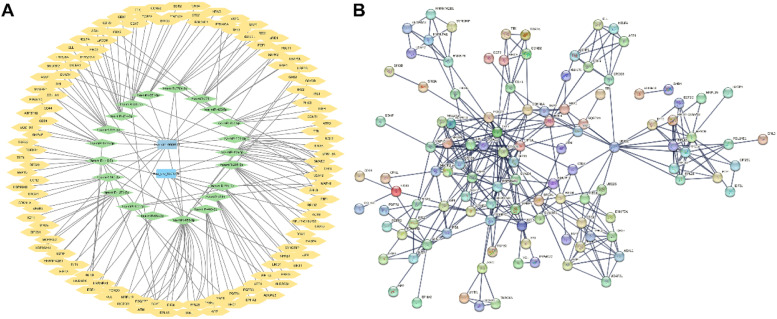
circRNA-miRNA-mRNA ceRNA network and PPI interaction network construction. (A) circRNA-miRNA-mRNA ceRNA network. (B) PPI interaction network.

After the enrichment and annotation of all the DEcircRNA-related miRNAs overlapped with GSE154255, a total of 60 breast cancer-associated miRNAs were obtained. The 122 downstream DEmRNAs were obtained with the help of StarBase, STRING, Cytoscape, and GSE52194. Finally, the ceRNA network with 2 circRNAs, 44 miRNAs, and 122 mRNAs was constructed and visualized (Fig. 3A). Then the downstream mRNAs were enrolled in the PPI interaction network using STRING database with high confidence 0.700 and disconnected nodes hidden in the network (Fig. 3B). The PPI network includes 122 number of nodes, 338 number of edges, 5.54 average node degree, and PPI enrichment p-value < 1.0e-16.

### GO and KEGG enrichment bubble diagrams

**Fig. 4:**
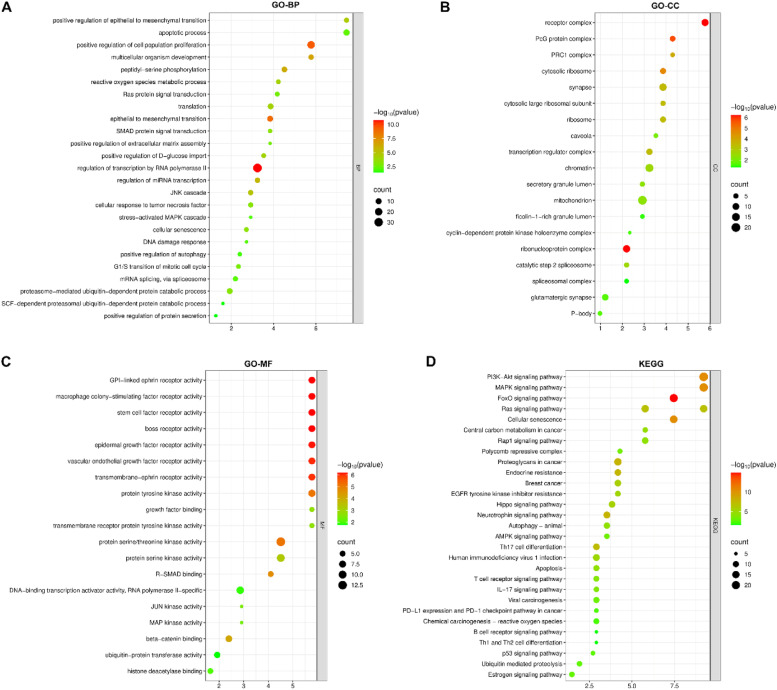
The GO and KEGG enrichment of circRNA-associated genes. (A‒C) The categories of Biological Process (BP) (A), Cellular Component (CC) (B), and Molecular Function (MF) (C) of GO enrichment results. (D) The KEGG enrichment analysis data.

All the mRNAs in the ceRNAs were annotated in the GO and KEGG enrichment analysis using the DAVID database. Among the categories of Biological Process (BP), Cellular Component (CC), and Molecular Function (MF), the top three enriched GO terms for each category were “regulation of epithelial to mesenchymal transition, apoptotic process, and regulation of cell proliferation”, “receptor complex, PcG protein complex, and PRC1 complex”, and “GPI-linked ephrin receptor activity, macrophage colony-stimulating factor receptor activity, and stem cell factor receptor activity”, respectively (Figs. 4A‒C). The top three enriched KEGG terms were the PI3K-Akt signaling pathway, the MAPK signaling pathway, and the FoxO signaling pathway (Fig. 4D).

### Expression and value of hsa_circ_0089131/miR-378a-3p/TP53 validation

**Fig. 5:**
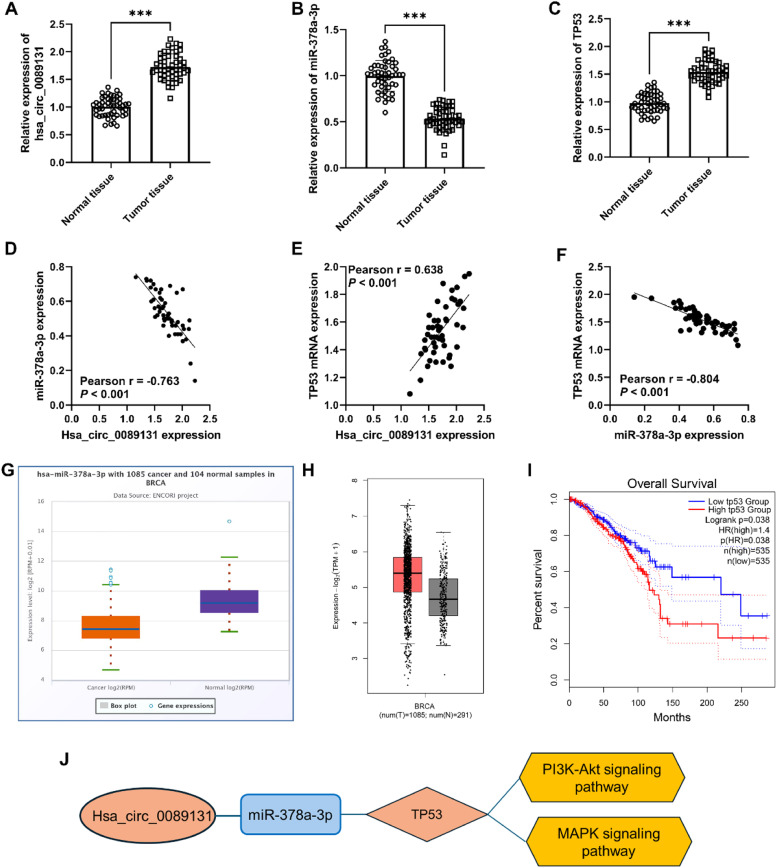
Expression and value of hsa_circ_0089131/miR-378a-3p/TP53 validation in breast cancer. *** *p* < 0.001. (A) hsa_circ_0089131 expression was upregulated in breast cancer tissues. (B) miR-378a-3p levels were decreased in cancer tissues. (C) An increased expression of TP53 mRNA was observed in breast cancer tissues. (D‒F) The correlation among hsa_circ_0089131, miR-378a-3p, and TP53. (G) StarBase database was used to validate miR-378a-3p expression in breast cancer. (H and I) GEPIA2 database was utilized to validate TP53 expression (H) and its prognostic value (I). (J) hsa_circ_0089131-miR-378a-3p-TP53-PI3K-Akt/MAPK signaling pathway network was constructed.

The RT-qPCR was utilized to measure the expression of hsa_circ_0089131/miR-378a-3p/TP53 in breast cancer tissues. The results indicated that hsa_circ_0089131 and TP53 mRNA expression were upregulated, while miR-378a-3p expression was decreased in breast cancer tissues (Figs. 5A‒C). The Pearson correlation analysis showed a negative correlation between hsa_circ_0089131 and miR-378a-3p expression in tissues and a positive correlation between hsa_circ_0089131 and TP53 mRNA, as well as a negative correlation between miR-378a-3p and TP53 mRNA levels (Figs. 5D‒F). Then, the decreased miR-378a-3p expression in Breast Cancer (BRCA) was verified in the ENCORI project (StarBase, Fig. 5G). However, miR-378a-3p did not show prognostic value in breast cancer (data is not shown). The GEPIA database was utilized to validate the expression and prognostic value of TP53 in breast cancer. The TP53 expression was increased in breast cancer (Fig. 5H) and high TP53 expression was related to poor overall survival rate in breast cancer patients (Fig. 5I). Based on the KEGG enrichment results, TP53 was involved in the PI3K-Akt signaling pathway and the MAPK signaling pathway. Thus, the hsa_circ_0089131-miR-378a-3p-TP53-PI3K-Akt/MAPK signaling pathway ceRNA network was constructed (Fig. 5J).

## Discussion

Malignant tumor is a kind of multi-factor participation, an extremely complex disease, and breast cancer has long been a female malignant tumor that cannot be ignored.^1^^,^^13^ People are committed to seeking biomarkers that can contribute to early detection, precise diagnosis and treatment, and improvement of survival.^14^^,^^15^ In this study, the authors comprehensively analyzed the breast cancer-related data from the GEO database, aiming to reveal the differential expression of circRNAs, miRNAs, and mRNAs, and construct the ceRNA network of breast cancer. This line of work has provided key clues for a deeper understanding of the molecular mechanisms of breast cancer.

Through comprehensive analysis of multiple circRNA-related GEO databases, the authors successfully screened two metastasis-related DEcircRNAs, namely hsa_circ_0001936 and hsa_circ_0089131. The finding is significant because metastasis is one of the main causes of poor prognosis in breast cancer patients, and identifying circRNAs associated with metastasis helps us to explore the molecular pathways of breast cancer metastasis. For example, hsa_circ_0089131 was verified to be upregulated in breast cancer tissues in subsequent experiments, suggesting that it may play a positive role in the progression of breast cancer. The differentially expressed hsa_circ_0001936 was also detected in lung cancer tissues, although it showed the opposite expression between microarray and RT-qPCR, hsa_circ_0001936 might act as a potential biomarker in lung cancer in Xuanwei patients.^16^ The hsa_circ_0001936 expression of microarray data in breast cancer also displayed inconsistent expression patterns.

After enrichment annotation of all miRNAs related to DEcircRNAs and cross-alignment with the GSE154255 database, a total of 60 miRNAs related to breast cancer were obtained. These miRNAs constitute an important part of the ceRNA network. As key regulators of gene expression, miRNAs are involved in a variety of biological processes during the occurrence and development of breast cancer.^17^ Previous studies reported prognosis-related, epithelial-mesenchymal transition-related circRNA-miRNA-mRNA ceRNA regulatory networks in breast cancer.^18^^,^^19^ In the current study, by constructing a metastasis-related ceRNA network containing 2 circRNAs, 44 miRNAs, and 122 mRNAs, the authors were able to visually observe the complex interaction between these molecules. The construction of this metastasis-related network provides a comprehensive framework for further studies on the molecular regulatory mechanisms of breast cancer.

GO enrichment analysis revealed key roles of breast cancer-related molecules in biological processes, cellular composition, and molecular function. For example, among biological processes, “regulation of epithelial-mesenchymal transition, apoptosis processes, as well as regulation of cell proliferation” is highly enriched, and these processes are closely related to metastasis and progression of breast cancer.^20^^,^^21^ In terms of cellular composition, the enrichment of “receptor complexes, PcG protein complexes, and PRC1 complexes” suggests that these structures are important in the biological behavior of breast cancer cells. PcG protein complexes (the best-characterized complexes are PRC1 and PRC2) played crucial roles in transcriptional silencing.^22^^,^^23^ In terms of molecular function, enrichment of “GPI-linked Ephrin receptor activity, macrophage colony-stimulating factor receptor activity, and stem cell factor receptor activity” suggested the key role of related molecules in breast cancer cell signaling. KEGG pathway enrichment analysis showed that breast cancer-related mRNAs were significantly enriched in AKT/PI3K and MAPK signaling pathways. The AKT/PI3K signaling pathway is involved in multiple processes such as cell survival, proliferation, metabolism, and angiogenesis, and its abnormal activation plays a key role in the occurrence, development, and metastasis of breast cancer.^24^ The MAPK signaling pathway plays an important role in cell growth, differentiation, apoptosis, and stress response, and is often dysregulated in breast cancer.^25^ Enrichment of these pathways provides potential molecular targets for breast cancer treatment, and it is expected to develop new therapeutic strategies by intervening in these pathways.

Through a variety of databases and experimental methods, the expression and correlation between hsa_circ_0089131, miR-378a-3p, and TP53 in breast cancer were verified. Consistently, decreased miR-378a-3p was also reported in breast cancer and was related to poor prognosis of breast cancer patients treated with tamoxifen in previous studies.^26^^,^^27^ Different mutations in the TP53 gene have been reported in breast cancer and revealed its important role in mammary carcinogenesis.^28^^,^^29^ The Current study indicated the prognostic value of TP53 in breast cancer using the GEPIA database, which is based on the data from the TCGA and the GTEx projects. A previous study demonstrated that TP53 could predict an unfavorable prognosis in TNBC patients.^30^ Based on KEGG enrichment results, the ceRNA network of hsa_circ_0089131-miR-378a-3p-TP53-PI3K-Akt /MAPK signaling pathway was constructed. The construction of this network reveals the complex regulatory relationships among circRNA, miRNA, and mRNA, which provide important clues for understanding the molecular mechanisms of breast cancer metastasis and prognosis.

The above circRNA-miRNA-mRNA ceRNA network model was constructed based on Chinese population data, whose core goal is to explore potential molecular markers that can be used to evaluate the metastasis and prognosis of breast cancer. The present study is still at the level of bioinformatic analysis. In the next step, the authors plan to combine domestic multi-center clinical resources to carry out clinical validation of the selected key molecular markers in large samples. If the validation results are ideal, the authors will cooperate with the biotechnology team to develop convenient detection methods based on these markers (such as RT-qPCR detection kits). In view of the high incidence of metastatic breast cancer in developing countries, future plans will be combined with health economic models to evaluate the cost-effectiveness of the test in early warning of metastasis and reducing the expenditure on late treatment, so as to provide data support for public health decision-making.

However, this study has certain limitations. Although the ceRNA network was constructed and some molecular expressions were verified, these results need to be further verified in more clinical samples and functional experiments in vitro and in vivo. Firstly, the clinical significance of hsa_circ_0089131, miR-378a-3p, and TP53 in breast cancer was obtained from an online database, thus, their clinical role needs to be verified using a cohort study with follow-up survival data. Secondly, the current study did not address the association of ctDNA with mutations. This direction can be an important extension for future research, for example, combining databases or clinical samples containing multi-omics data (transcriptome + ctDNA + mutation profile), to further explore the cross-regulation mechanism between the ceRNA network and ctDNA/mutation, so as to reveal the metastasis and prognosis mechanism of breast cancer more comprehensively. Additionally, the regulatory mechanism between hsa_circ_0089131, miR-378a-3p, and TP53, as well as their effects on biological functions such as proliferation, migration, and invasion of breast cancer cells, were further studied through cell and animal experiments.

## Conclusion

In conclusion, this study constructed the hsa_circ_0089131-miR-378a-3p-TP53-PI3K-Akt/MAPK signaling pathway ceRNA regulatory network related to breast cancer metastasis and preliminarily clarified the important role of hsa_circ_0089131, miR-378a-3p and TP53, which laid a bioinformatic foundation for an in-depth understanding of the molecular mechanism of breast cancer. The ceRNA network may provide a theoretical basis for subsequent cell function and animal experiments, which illustrate a complex regulatory mechanism of breast cancer.

## Authors’ contributions

Hongming Song and Haibo Wang carried out the concepts, design and definition of intellectual content, Mingkai Gong, Xiangping Liu, Haiyan Hu, Wenfeng Li, Chen Liu, Zhongliang Ma, Yuanxiang Wang and Bing Du provided assistance for data acquisition, data analysis and statistical analysis. All authors performed the experiment and drafted the manuscript. Hongming Song and Haibo Wang revised the manuscript critically for important intellectual content. All authors have read and approved the content of the manuscript.

## Funding

This work was funded by the 10.13039/501100014761Qingdao Natural Science Foundation (Grant No. 25–1–1–224-zyyd-jch) and the China Anti-Cancer 10.13039/100008233Association Foundation（Grant No. CETSDCORP2772-02) .

## Declaration of competing interest

The authors declare no conflicts of interest.

## Data Availability

Data Availability: The datasets generated and/or analyzed during the current study are available from the corresponding author upon reasonable request.
